# RTX-Toxins

**DOI:** 10.3390/toxins12060359

**Published:** 2020-05-30

**Authors:** Roland Benz

**Affiliations:** 1 Department of Life Sciences and Chemistry, Jacobs University Bremen, Campusring 1, 28759 Bremen, Germany; R.Benz@jacobs-university.de; 2 Rudolf-Virchow-Center for Experimental Biomedicine, University of Würzburg, Versbacher Str. 9, 97078 Würzburg, Germany

RTX-Toxins (Repeats in ToXin) are members of a rapidly expanding family of proteins [1]. Characteristic for them is the presence of multiple sequences of nonapeptide repeats of the consensus sequence G-G-X-G-(N/D)-D-X-(L/I/F)-X (where X can be any amino acid) in the N-terminal half of the proteins [2,3]. The RTX toxins are virulence factors that are synthesized by a diverse group of Gram-negative pathogens [3]. All members of the RTX toxin family share common gene organization and distinctive structural features. Although variations do exist, the typical RTX toxin operon consists of four different genes that are designated *rtxC*, *A*, *B*, and *D* in transcriptional order [3,4]. *rtxA* codes for the structural component of the toxin molecules. They are transported from the cytoplasm to the cell surface by a type one secretion system (T1SS), composed of the gene products RtxB and RtxD [5,6]. For export across the outer membrane, the inner membrane ATP-dependent export machinery, composed of RtxB (ATPase) and RtxD (linker protein), needs the help of an outer membrane channel in the TolC-family [5,6,7]. The corresponding gene is, in certain cases, part of the RTX operon [7]. Most RTX toxins require post-translational modification to become biologically active. This is performed by RtxC, which is an acyl carrier protein that binds fatty acids to one or two specific lysines of RtxA by acylation [8,9]. The export signal for the transport of RtxA to the cell surface is localized at the C-terminal end of RtxA and comprises about 60 amino acids [10].

RtxA toxins normally contain less than ten repeats and at maximum up to 45 repeats (CyaA of *Bordetella pertussis*) [11]. They are able to bind calcium ions with high affinity to form a special structure where each repeat forms a half-site of the β-roll motif essential for target cell recognition [12,13]. The calcium ions are in contact with the aspartate in the sixth position of the nonapeptide. RTX proteins comprise many different functional categories. Well known of these is the RTX cytolysin family, where HlyA of uropathogenic *Escherichia coli* may represent the classical and well-studied example of a pore-forming RTX toxin [14]. Besides the cytolysins as pathogenicity factors, the RTX proteins also contain subfamilies of many diverse functions. These subfamilies are consisting of RTX adhesins, enzymatic toxins, bacteriocins, surface layer proteins, and hydrolytic enzymes, such as proteases and lipases [1]. More than 1000 RTX proteins have been identified to date [1]. RTX proteins can be very large and may comprise more than 6000 amino acids [15]. RTX toxins are much smaller and contain between 1000 and 2000 amino acids [1,2,3,14,16,17]. Some RTX toxins, like the HlyA toxin of *E. coli*, act on a wide range of different host cell types, but others, e.g., the leukotoxins of *A. actinomycetemcomitans*, act only on a restricted number of cells in a species-specific fashion [18].

This Special Issue focuses on the function of RTX toxins of different subfamilies and their role in disease [16,17,18,19,20,21,22]. Of special interest is the structure of the nonapeptide repeats and their interaction with calcium ions, which represents some prerequisites for target cell recognition [13]. Calcium ions are tightly bound to the repeats, which form parallel β-rolls only in the presence of calcium ions and cannot form without them because they are integral parts of the structure. The reason for the RTX domains is presumably related to the secretion and folding of the RTX proteins. In low calcium, cytoplasmic fluid, the repeat domains are unfolded and become folded in high calcium external fluid, which means that the repeats could act as chaperones [13]. On the other hand, it is clear that calcium-mediated conformation changes of the repeats play an important role in target cell recognition and in membrane-activity of certain RTX toxins [13,17]. This is not only the case for pore formation of cytolytic RTX toxins but also for the AMS8 lipase from the Antarctic *Pseudomonas fluorescens* strain AMS8 [23]. The three RTX motifs of this protein react to the presence of calcium ions. The tightly bound calcium results in an increase of lipase activity together with changes in its secondary structure [23].

Besides the nonapeptide repeats and the acylation of the ε-amino groups of certain lysines with fatty acids, RTX toxins also need cell-surface receptors for target cell recognition and invasion. Studies with different RTX toxins, such as LtxA (*Aggregatibacter actinomycetemcomitans*), LktA (*Mannheimia haemolytica*), CyaA (*Bordetella pertussis*), HlyA (uropathogenic *Escherichia coli*), and ApxIII (*Actinobacillus pleuropneumoniae*), demonstrated that a single host cell receptor is responsible for RTX toxin cell interactions [18,22]. These are the β_2_ integrins, which are composed of heterodimeric members with four unique alpha subunits and a single beta subunit. β_2_ integrins are only found on leukocytes, including neutrophils and monocytes, the first responders to inflammation following bacterial infection, although the RTX toxins may also cause hemolysis caused by the formation of pores in red blood cells [24]. LtxA acts as a virulence factor for the oral pathogen *A. actinomycetemcomitans* causing aggressive periodontitis [18]. LtxA also binds to the β_2_ integrin lymphocyte function-associated antigen-1 (LFA-1; CD11a/CD18) on white blood cells (WBCs), causing cell death. LtxA (trade name Leukothera^®^) may provide therapeutic applications for the treatment of hematological malignancies and immune-mediated diseases [18]. All of the mentioned cytolysins form cation-selective pores in the cell membranes as the primary event [16,17,18,20,21,24,25]. This means that antibodies directed against specific RTX toxins could protect from diseases [21]. RTX toxins could, therefore, serve as antigens for vaccine research and development to treat the corresponding bacterial diseases [20,21,22]. These considerations are important for diagnostic applications in human and veterinary medicine, as outlined in three contributions in this Special Issue [18,21,22].

Enterohemolysin (EHEC-hemolysin, EHEC-Hly), which belongs to the RTX toxin family, is besides Shiga toxin (Stx), a potential virulence factor of enterohemorrhagic *E. coli* (EHEC). This disease is frequently associated with hemorrhagic colitis (HC) and hemolytic uremic syndrome (HUS) [20]. A review in this Special Issue describes the current knowledge of EHEC-Hly together with the influence of various regulator proteins on its production and discusses the diagnostic role of EHEC-Hly. It also sheds light on the different mechanisms leading to damage of target cells together with the putative genetic evolution of the toxin [20].

The RTX toxin CyaA produced in *B. pertussis* is the causative agent of whooping cough [3,7,19,26]. The toxin contains different domains. The N-terminal domain of 400 amino acids has calcium- and calmodulin-dependent adenylyl cyclase activity. The toxin has the capacity to translocate this domain directly across the plasma membrane into the cytosol of eukaryotic cells. After translocation, this leads to uncontrolled production of cAMP, intoxicating the target cell. The exact mechanism is not known, but it has been claimed in a previous study that CyaA exhibits a phospholipase A (PLA) activity that could be involved in the transport of the adenylyl cyclase across the cell membrane [27]. In order to clarify the potential role of CyaA as PLA, a study in the Special Issue investigated two different samples of CyaA for phospholipase activity [26]. In this study, no PLA activity associated with both batches of CyaA could be found [26].

CyaA acts on target cells by uncontrolled production of cyclic adenosine monophosphate (cAMP) in the presence of calmodulin [7,8,9,17,19]. Similarly, the edema toxin (ETX) of *Bacillus anthracis* also produces calmodulin-dependent cAMP, which means that both toxins lead to a massive increase of this second messenger [19]. Another study of the Special Issue deals with the opsonophagocytic killing of invading pathogens by myeloid phagocytes [19]. This study recognized that CyaA has a much stronger effect on activation and phosphorylation of Syk, Vav, and Pyk2, thus inhibiting opsonophagocytosis because CyaA rapidly penetrates cells directly across their plasma membrane [19]. On the other hand, the ETX-generated cAMP gradient only poorly inhibited the activation and phosphorylation of these signaling proteins because the slow cellular entry of ETX depends on receptor-mediated early endosomal pathway [19]. This means that differences in the spatiotemporal distribution of cAMP produced by CyaA and ETX differentially affect the opsonophagocytic signaling in myeloid phagocytes [19].

In the past, it has been discussed whether the T1SS system for secretion of RTX proteins can be used for the production and secretion of foreign proteins in bacterial cells [28]. The minimum requirement for this is the attachment of the RTX protein export signal to their C-termini [28]. Similarly, it has been investigated if the shortcut of the CyaA-mediated transport of the adenylyl cyclase activity into target cells could be used for antigen-delivery into immune cells [29]. For this, the CyaA has to be detoxified by the insertion of foreign antigenic determinants into the adenylyl cyclase domain [29]. A severe problem for the use of recombinant fatty-acylated RTX cytolysins from inclusion bodies produced in *E. coli* is the presence of high amounts of *E. coli* lipopolysaccharide (LPS or endotoxin) [29,30]. A contribution to this Special Issue describes a simple procedure for the purification of RTX cytolysins from LPS [30]. This procedure is based on the complete unfolding of the toxins in 8 M urea, followed by their binding to a chromatographic medium. Contaminating LPS is removed by an extensive wash of the column with urea and detergent [30]. Finally, residual detergent is removed by a wash with 8 M urea, and the RTX protein can be eluted from the column. The authors of the study describe the application of this method to four RTX cytolysins, the *Bordetella pertussis* CyaA and the hemolysins of *Escherichia coli* (HlyA), *Kingella kingae* (RtxA), and *Actinobacillus pleuropneumoniae* (ApxIA) [30].
